# Emergence of the Zoonotic Biliary Trematode *Pseudamphistomum truncatum* in Grey Seals (*Halichoerus grypus*) in the Baltic Sea

**DOI:** 10.1371/journal.pone.0164782

**Published:** 2016-10-18

**Authors:** Aleksija S. Neimanis, Charlotta Moraeus, Anders Bergman, Anders Bignert, Johan Höglund, Karl Lundström, Annika Strömberg, Britt-Marie Bäcklin

**Affiliations:** 1 Department of Pathology and Wildlife Diseases, National Veterinary Institute, Uppsala, Sweden; 2 Department of Environmental Research and Monitoring, Swedish Museum of Natural History, Stockholm, Sweden; 3 Department of Biomedical Science and Veterinary Public Health, Section for Parasitology, Swedish University of Agricultural Sciences, Uppsala, Sweden; 4 Department of Aquatic Resources, Swedish University of Agricultural Sciences, Lysekil, Sweden; Universidade de Aveiro, PORTUGAL

## Abstract

The biliary trematode *Pseudamphistomum truncatum* parasitizes a wide range of fish-eating mammals, including humans. Here we report the emergence of this parasite in grey seals (*Halichoerus grypus*) in the Baltic Sea. One hundred eighty-three of 1 554 grey seals (11.9%) examined from 2002–2013 had detectable hepatobiliary trematode infection. Parasite identification was confirmed as *P*. *truncatum* by sequencing the ITS2 region of a pool of five to 10 trematodes from each of ten seals collected off the coast of seven different Swedish counties. The proportion of seals parasitized by *P*. *truncatum* increased significantly over time and with increasing age of seals. Males were 3.1 times more likely to be parasitized than females and animals killed in fishery interactions were less likely to be parasitized than animals found dead or hunted. There was no significant difference in parasitism of seals examined from the Gulf of Bothnia versus those examined from the Baltic Proper. Although the majority of infections were mild, *P*. *truncatum* can cause severe hepatobiliary disease and resulted in liver failure in at least one seal. Because cyprinid fish are the second intermediate host for opisthorchiid trematodes, diets of grey seals from the Baltic Sea were analysed regarding presence of cyprinids. The proportion of gastrointestinal tracts containing cyprinid remains was ten times higher in seals examined from 2008 to 2013 (12.2%) than those examined from 2002 to 2007 (1.2%) and coincided with a general increase of trematode parasitism in the host population. The emergence and relatively common occurrence of *P*. *truncatum* in grey seals signals the presence of this parasite in the Baltic Sea ecosystem and demonstrates how aquatic mammals can serve as excellent sentinels of marine ecosystem change. Investigation of drivers behind *P*. *truncatum* emergence and infection risk for other mammals, including humans, is highly warranted.

## Introduction

The grey seal (*Halichoerus grypus*) population in the Baltic Sea has suffered numerous insults including excessive hunting in the beginning of the 20^th^ century followed by reproductive failure from polychlorinated biphenyls (PCBs) and other environmental contaminants in the latter half of the century [1]. Although the population has recovered in recent years, it is still far from historic levels [1]. As part of an on-going monitoring program to investigate health and environmental contaminants in grey seals from the Swedish Baltic coast, approximately 100–150 animals that have been hunted, incidentally caught in fishing gear, or found stranded are examined by the Swedish Museum of Natural History each year.

The biliary trematode, *Pseudamphistomum truncatum*, has an indirect life cycle, which includes a broad range of fish-eating mammals as the definitive host. Although the life cycle in the Baltic environment is poorly understood, opisthorchiid trematodes typically have two intermediate hosts: freshwater gastropods and cyprinid fish [2]. Roach (*Rutilus rutilus*) are suspected to play a key role as metacercariae of *P*. *truncatum* have been demonstrated in this fish species in Germany, Denmark and Ireland [3,4,5]. Previously described in marine mammal species (Caspian seals, *Pusa caspisca*) [6,7] and various terrestrial and semi-aquatic mammals such as wild mustelids and canids [8,9,10,11], cats (*Felis domesticus*) [12] and even humans [13] in Asia and Europe, this trematode recently also has emerged in otters (*Lutra lutra)* and American mink (*Mustela vison*) in Great Britain and Ireland [14,5].

In Sweden, *P*. *truncatum* was first recorded in grey seals from the Baltic in 1986, however only isolated reports exist in the archives of the Swedish Museum of Natural History prior to 2002 [15]. Here we provide evidence that this trematode recently has emerged in this population of grey seals, can impair seal health and is relatively common in the Baltic ecosystem. These results demonstrate how marine mammals can serve as excellent sentinels of changes in marine ecosystems and provide additional data on geographic and species range expansion of this zoonotic parasite.

## Materials and Methods

### Animals

**Fig 1:**
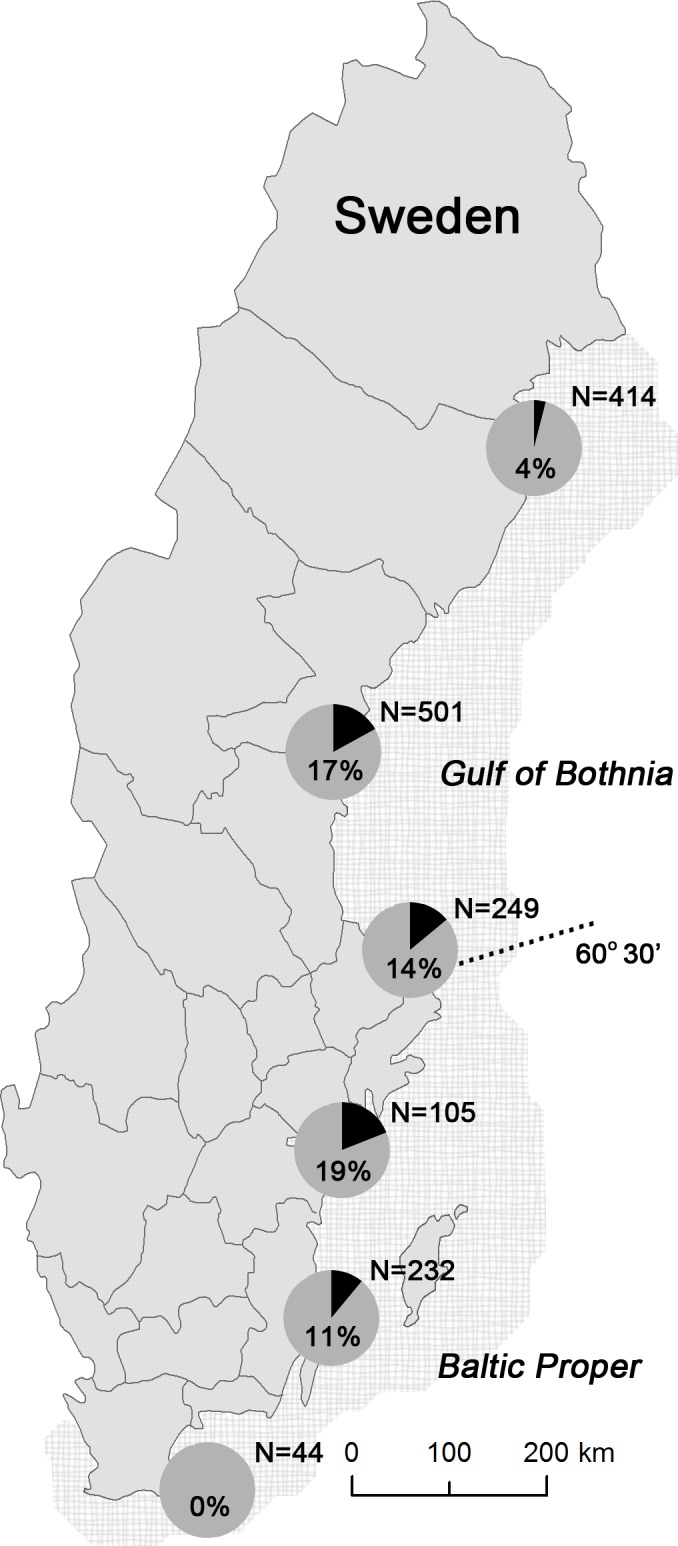
Map of Sweden indicating where grey seals (*Halichoerus grypus*) were collected showing the proportion of seals infected with *Pseudamphistomum truncatum* from 2002–2013. Data are grouped to represent two adjacent counties at once. Pie charts illustrate the proportion of infected seals per total number of animals examined from each pair of counties.

From 2002 to 2013, carcasses or a suite of standard tissues were collected from 1,554 grey seals from the Swedish Baltic Sea and examined by the Swedish Museum of Natural History. Animals died in fishery interactions (n = 562), were hunted (n = 930) or were sick or found dead (n = 62). Carcasses and tissues were examined and sampled following a standard protocol [16]. Location, date and cause of death were recorded for each animal. Animals were collected off the entire Baltic coast of Sweden, from Norrbotten in the north to Skåne in the south, and also included waters around the islands of Gotland and Öland (Fig 1). Location data were not available for 16 seals. Seals were collected from the Baltic Proper (south of N 60°30’) (n = 549) and the Gulf of Bothnia (north of N 60°30’) (n = 989) sea basins. Although animals were collected year round, there were seasonal and spatial sampling biases for hunted seals and seals that died in fishery interactions. Hunting is limited to mid-April until the end of December and numbers of hunted seals consistently were highest in May when hunting was facilitated by ice. Numbers of seals that died in fishery interactions were greatest in the autumn reflecting greatest fishing effort. More animals were hunted than died in fishery interactions in the Gulf of Bothnia whereas the opposite was true for the Baltic Proper. Biological data routinely recorded included length, sex and reproductive status. A total of 836 animals were males, 712 were females and six were of unknown sex. Animals were aged by sectioning of a lower canine tooth and counting of cementum annuli according to Johnston and Watt [17]. Age ranged from 0 to 40 years but could not be determined for 33 animals because teeth were not available.

The Swedish Museum of Natural History works under the directive of the Swedish Environmental Protection Agency (Swedish EPA contract number 2213-16-0004) to receive grey seal carcasses and tissues from dead grey seals for the monitoring of environmental contaminants and seal health status. No live animals or tissues from live animals were used in this study, therefore no permit from an animal ethics committee was required. Samples came from seals found dead, from seals incidentally drowned in fishing gear, and from seals that were hunted within the strictly regulated protective hunt on grey seals associated with fishing gear authorized by the Swedish Environmental Protection Agency.

### Investigation of trematode infection

The liver and portal lymph node from each seal were weighed and these tissues along with the gall bladder and pancreas were examined for the presence of trematodes and associated pathological lesions. Extent of liver involvement and qualitative assessment of degree of infection and associated lesions were recorded. Any additional extrahepatobiliary lesions, regardless of cause, also were noted. Parasites were stored in 70% ethanol and tissues from a subset of seals were fixed in neutrally buffered formalin for microscopic examination. Formalin-fixed tissues were processed for routine histological examination. Briefly, tissues were dehydrated in graded alcohol, cleared in Histolab-Clear®, and embedded in paraffin. Sections (5 μm) were stained with Mayer’s hematoxylin and eosin [18].

### Genotyping of trematodes

Trematodes collected from the livers of ten grey seals sampled from 2003 to 2010 from the Gulf of Bothnia (Uppsala, Gävleborg, Norrbotten and Västernorrland counties) and the Baltic Proper (Södermanland, Östergötland and Kalmar counties) were genotyped based on sequence information in the internal transcribed spacer region 2 (ITS2) within the ribosomal DNA (rDNA) gene array. Briefly, the ITS2 region was amplified from genomic DNA prepared from ten pooled samples of 5–10 worms from each seal, by polymerase chain reaction (PCR) using the primer combinations as described in Skov et al. [4]. After PCR amplifications, products were run on agarose gels, cleaned with the BioRad kit and subsequently sent for sequencing. The quality of the obtained sequences was first checked and analyzed using the software CLC Main Workbench v5.6.1, before sequences were identified and submitted for nucleotide search using BLASTN 2.2.31 in NCBI PubMed [19].

### Diet analysis

To investigate the presence of cyprinid fish in the diet of grey seals in the Baltic Sea, gastrointestinal tracts containing prey items were collected from 572 seals hunted from 2002–2013 in the Baltic Proper (n = 146) and the Gulf of Bothnia (n = 426). Animals killed in fishery interactions or found sick or dead were not included in this analysis because of potential bias from recent feeding in fishing gear or illness [20].

Determination of prey-species composition using prey hard parts was carried out as described in Lundström et al. [21]. Cyprinid fish were identified to family level based on otoliths, pharyngeal teeth and chewing pads. The cyprinid frequency of occurrence was calculated as the number of seals containing cyprinid remains, using all hard-part structures, relative to the total number of seals containing prey.

### Statistical analyses

Statistical analyses were performed to investigate temporal changes in parasite prevalence and dietary habits, and to determine the influence of explanatory variables possibly associated with parasitism. Multivariable logistic regression and Chi-square analysis were carried out using the software package PIA [22] and Microsoft Excel, 2010 (Microsoft Corporation), respectively.

Multivariable logistic regression [23] was performed to investigate the influence of year, age, sex, sea basin and cause of death on the presence or absence of trematodes (S1 Appendix). Various models were compared using the Akaike information criteria (AIC) and the final model was selected using backwards selection. An interaction term between sex and age (sex * age) initially was included in the model and gave a slightly better fit but lead to non-significant individual influence of both sex and age and was therefore not included in the final model. The significance of each variable was checked by the Wald test statistic and the odds ratios with their corresponding 95% confidence intervals were calculated.

The age, sex ratio and the cause of death of the sampled seals were fairly constant over the study period whereas the proportion of seals collected in the Baltic Proper increased over time compared to those collected in the Gulf of Bothnia.

Temporal changes in dietary habits were examined using a Chi-square test. Prevalence of trematodes increased dramatically in 2008 so this analysis was performed to see if occurrence of cyprinid hard parts in the grey seal gastrointestinal tracts also increased. The data set was divided into two periods (2002–2007, n = 334 and 2008–2013, n = 238, S2 Appendix). The proportion of seals with cyprinid hard parts in the gastrointestinal tract was compared between the two time periods to test the null hypothesis that the proportions did not differ.

## Results

### Trematode infection

**Fig 2:**
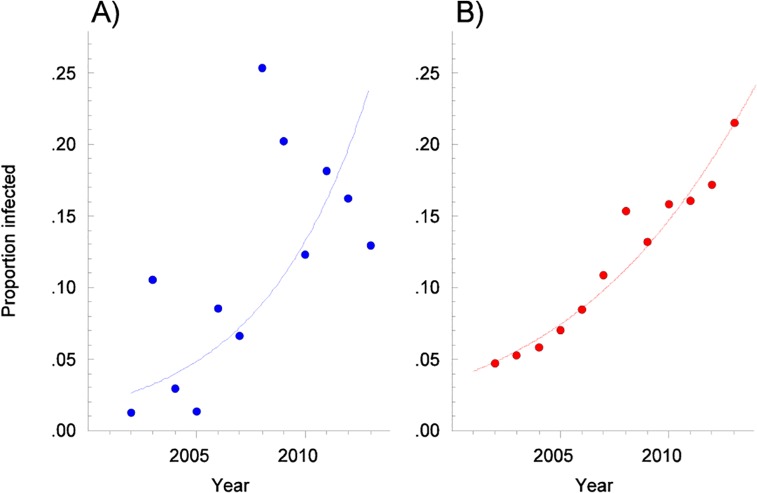
A) Proportion of grey seals (*Halichoerus grypus*) from the Baltic coast of Sweden infected with *Pseudamphistomum truncatum* per year from 2002 to 2013 unadjusted for explanatory variables. The proportion of infected seals increases significantly over time (p<0.01, log-linear regression). B) Predicted proportion of grey seals from the Baltic coast of Sweden infected with *P*. *truncatum* per year from 2002 to 2013, adjusted proportions using an average of 8 years of age and observed mean values for sex and cause of death. This reduces the variance in the logistic model (p< 0.001, full logistic model compared to intercept only).

Trematodes were observed in the hepatobiliary system of 183 of 1,554 seals (11.9%). The proportion of infected seals of those examined each year ranged from 1.2% in 2002 to 26.2% in 2008 (Fig 2). Prevalence continued to be 12.0% or greater in seals examined from 2009 to 2013. Trematodes were more frequently found in males (137/836 or 16.4% of all males examined) than females (45/712 or 6.3% of all females examined) and young animals were underrepresented. Of all pups and 1-year olds examined (n = 478), 13 (2.7%) had detectable trematodes (Table 1). Infected seals were found off the coast of all counties bordering the Baltic Sea with the exception of the two southernmost counties, Skåne and Blekinge (Fig 1).

**Table 1:** Prevalence of *Pseudamphistomum truncatum* infection by age and sex class of grey seals (*Halichoerus grypus*) examined from the Swedish Baltic Sea 2002–2013.

Age (years)	Number of seals examined^a^	Number of seals with detectable trematodes^a^	Prevalence of infection per age and sex class Infected/Total (%)^a^
**0**	N = 397 (M = 213, F = 182, U = 2)	N = 9 (M = 5, F = 4, U = 0)	9/397 (2.3%) (M = 2.3%, F = 2.2%, U = 0%)
**1**	N = 81 (M = 34, F = 46, U = 1)	N = 4 (M = 1, F = 3, U = 0)	4/81 (4.9%) (M = 2.9%, F = 6.5%, U = 0%)
**2**	N = 138 (M = 76, F = 62, U = 0)	N = 15 (M = 8, F = 7, U = 0)	15/138 (10.9%) (M = 10.5%, F = 11.3%, U = 0%)
**3**	N = 130 (M = 75, F = 55, U = 0)	N = 13 (M = 8, F = 5, U = 0)	13/130 (10.0%) (M = 10.7%, F = 9.1%, U = 0%)
**4**	N = 103 (M = 46, F = 56, U = 1)	N = 11 (M = 8, F = 3, U = 0)	11/103 (10.7%) (M = 17.4%, F = 5.4%, U = 0%)
**5**	N = 82 (M = 47, F = 35, U = 0)	N = 9 (M = 8, F = 1, U = 0)	9/82 (11.0%) (M = 17.0%, F = 2.9%, U = 0%)
**6–10**	N = 274 (M = 136, F = 137, U = 1)	N = 51 (M = 36, F = 14, U = 1)	51/274 (18.6%) (M = 26.5%, F = 10.2%, U = 100%)
**11–15**	N = 181 (M = 111, F = 70, U = 0)	N = 33 (M = 29, F = 4, U = 0)	33/181 (18.2%) (M = 26.1%, F = 5.7%, U = 0%)
**16–20**	N = 91 (M = 55, F = 36, U = 0)	N = 18 (M = 17, F = 1, U = 0)	18/91 (19.8%) (M = 30.9%, F = 2.8%, U = 0%)
**>20**	N = 44 (M = 26, F = 18, U = 0)	N = 16 (M = 14, F = 2, U = 0)	16/44 (36.4%) (M = 53.8%, F = 11.1%, U = 0%)
**Age unknown**	N = 33 (M = 17, F = 15, U = 1)	N = 4 (M = 3, F = 1, U = 0)	4/33 (12.1%) (M = 17.6%, F = 6.7%, U = 0%)
**Total**	N = 1,544 (M = 836, F = 712, U = 6)	N = 183 (M = 137, F = 45, U = 1)	183/1,544 (11.9%) (M = 16.4%, F = 6.3%, U = 16.7%)

^a^N = total number of animals, M = male seals, F = female seals, U = seals of unknown sex.

**Fig 3:**
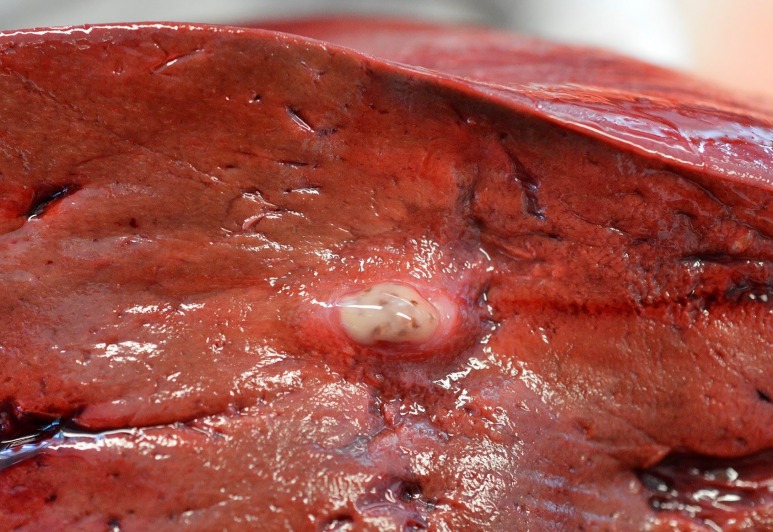
Cross section of the liver of a grey seal (*Halichoerus grypus*) with a mild, localized infection of *Pseudamphistomum truncatum*. Note the ectatic bile duct filled with beige exudate admixed with small, brown trematode parasites centrally. Bile duct walls are moderately thickened.

**Fig 4:**
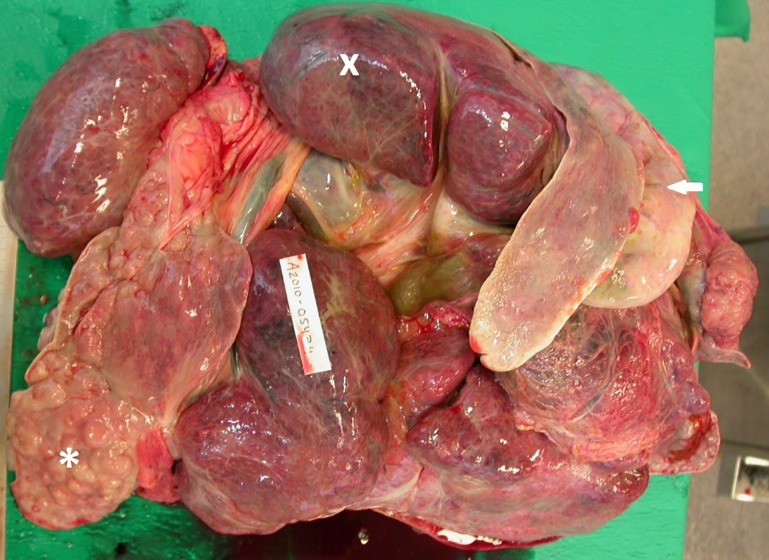
The liver of a grey seal (*Halichoerus grypus*) infected with *Pseudamphistomum truncatum* displaying chronic, severe cholangiohepatitis. Lobes vary from being markedly atrophic with only severely fibrotic and ectatic bile ducts and intervening connective tissue remaining (*) to being swollen and congested with evidence of hepatic fibrosis and necrosis on cut surface (x). The gall bladder is markedly enlarged and has thick walls (⇦).

Parasites were 1–1.5 mm long and typically were seen in intrahepatic bile ducts and/or in the gall bladder (Fig 3). Bile ducts associated with trematodes were prominent because they often were distended with thick, beige to yellow-brown material and walls were variably expanded by chronic inflammation and fibrosis (Fig 3). No parasites were detected in the pancreas. Parasitic infection ranged from mild and confined to a single liver lobe, to severe and widespread with involvement of the entire liver and gall bladder (Fig 4). An enlarged portal lymph node indicated the presence of trematodes.

**Fig 5:**
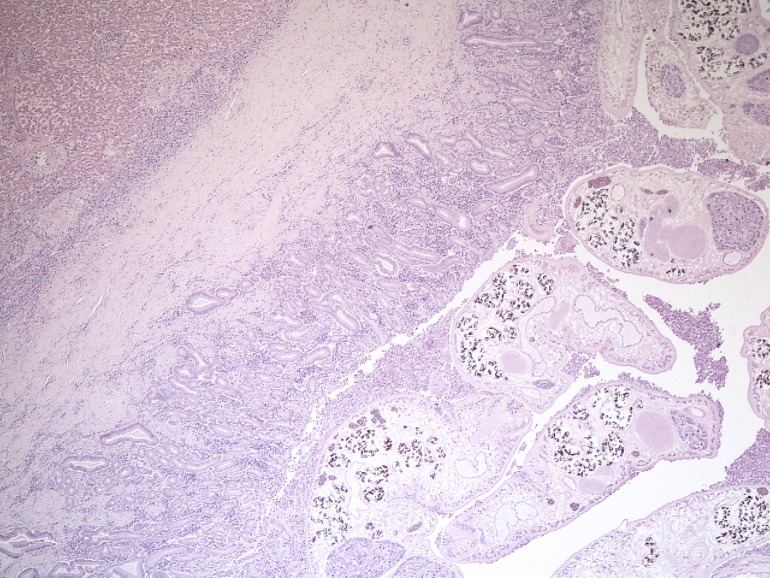
Photomicrograph of a major bile duct (right) and adjacent liver (left) of a grey seal (*Halichoerus grypus*) infected with *Pseudamphistomum truncatum*. The ductal mucosa is hyperplastic and the duct wall is thickened by fibrosis. Eosinophilic and lymphoplasmacytic inflammation extends through the mucosa, ductal wall and into the adjacent hepatic parenchyma, particularly in portal areas. Cross-sections of numerous adult trematodes are seen within the lumen of the duct. Magnification 40X.

Microscopically, trematodes had a spinous cuticle and their eggs were approximately 25 X 50 μm in size, operculated and had a thick, yellow-brown wall. Associated pathological changes consisted of variable eosinophilic and lymphoplasmacytic cholangiohepatitis, cholangitis and biliary fibrosis (Fig 5). In scattered, more affected areas, hepatic parenchyma was replaced by inflammatory and necrotic debris, often admixed with trematode eggs. In more severely parasitized animals (e.g. ≥100 adult flukes), hepatic architecture was effaced by necrosis, inflammation and/or fibrosis and the gall bladder wall was markedly thickened by inflammation, fibrosis and mucosal hyperplasia. Severe infection of thousands of trematodes led to liver failure in at least one individual based on effacement of normal liver parenchyma coupled with concurrent ascites and icterus. This 14 year old male seal was euthanized in 2010 in Östergötland county. Almost complete effacement of liver parenchyma but no icterus was seen in two other severely infected adult male seals that were found dead or were euthanized in 2005 and 2007.

### Sequence data

Sequencing results of ten pools of trematodes revealed a 338 bp region within the primer sequences. All sequences were identical apart from position 55, where there was a C instead of A in four pools, indicating the presence of two haplotypes. BLAST^®^ search of the consensus sequence revealed 100% identity to a sequence containing the complete ITS2 of *P*. *truncatum* (ID: gb|EU483072.1|). All sequences have been submitted to the European Nucleotide Archive (ENA) and accession numbers are pending.

### Diet analysis

**Fig 6:**
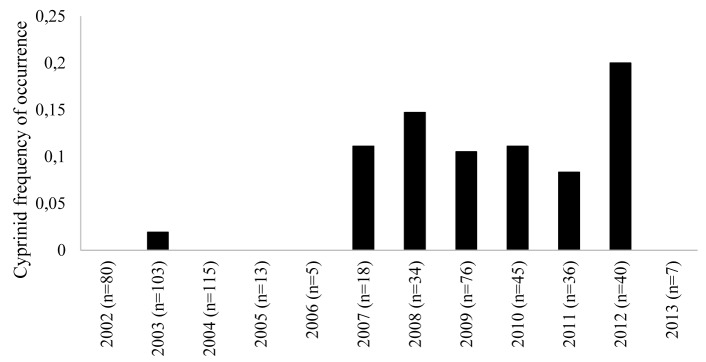
The frequency of cyprinid occurrence in the gastrointestinal contents of grey seals (*Halichoerus grypus*) collected from the Baltic Sea from 2002 to 2013. Annual frequencies were calculated by dividing the number of seals containing remnants of cyprinid fish by the total number of seals containing prey items per year. Sample sizes per year are shown in brackets.

From 2002 to 2007, cyprinid hard parts only were present in four of the 334 seals (1.2%) that contained prey items in their gastrointestinal tracts. From 2008 to 2013, 29 of the 238 seals (12.2%) containing prey items had cyprinid hard parts in their gastrointestinal tracts. The frequency of cyprinid occurrence over time is shown in Fig 6.

### Statistical results

In the final logistic regression model, year, sex and age were significant (p<0.0001), as was cause of death (p<0.001) (Table 2). Parasite prevalence increased significantly over time and with increasing age of seals. The 95% confidence interval for the odds ratio indicated that males were 2 to 4.5 times more likely to be parasitized compared to females (Table 2). Seals that were incidentally captured in fishing gear were less likely to be parasitized than seals hunted or found dead. Data are presented in Fig 2. A large part of the variation in prevalence can be explained by age, sex, year and cause of death, but taking these factors into account, prevalence of infection was still unexpectedly high in 2008.

**Table 2:** Results of multivariable logistic regression analysis to investigate the influence of the explanatory variables year, age, sex, cause of death and sea basin on parasitism of grey seals (*Halichoerus grypus*) with *Pseudamphistomum truncatum* in the Swedish Baltic Sea 2002–2013.

Variable^a^	Parameter estimate	Standard error	Wald test statistic	P-value	Estimated odds ratio	95% Confidence Interval for the odds ratio
**Intercept**	-19.7034	3.1258	39.734	0.0001	-	-
**Year**	0.1531	0.0286	28.604	0.0001	1.165	(1.102–1.233)
**Age**	0.0811	0.0124	42.803	0.0001	1.084	(1.058–1.111)
**Sex**	1.1247	0.1938	33.666	0.0001	3.079	(2.106–4.503)
**Cause of Death**	-0.5603	0.2062	7.382	0.0066	0.571	(0.381–0.855)

^a^Sea basin was not a significant variable and was removed from the final model.

The proportion of grey seals with cyprinid hard parts in their gastrointestinal tract was significantly greater in 2008–2013 (12.2%) than in 2002–2007 (1.2%) (p<0.0001).

## Discussion

According to the World Health Organization, an emerging disease has appeared in a population for the first time, or may have existed previously but is rapidly increasing in incidence or geographic range [24]. *Pseudamphistomum truncatum* was first recorded in Baltic grey seals in 1986, whereas only scattered cases were observed prior to 2002 [15]. Annual prevalence peaked in 2008 (26.2%), and although it decreased in subsequent years, it has continued to remain above 12% in seals examined by the Swedish Museum of Natural History. Thus, based on a highly significant increase in annual parasite prevalence from 2002 to 2013 it can be stated that *P*. *truncatum* is recently emerging in grey seals from Swedish waters of the Baltic Sea.

In addition to increased parasite prevalence over time, parasitism was significantly associated with sex, age and cause of death. Male seals were significantly more likely to be parasitized by trematodes than females (odds ratio = 3.1), which is consistent with previous findings of *P*. *truncatum* infection in Caspian seals [6], and other reports of male bias regarding parasitism in wild animals [25]. The underlying reasons for this sex bias in grey seals are unknown but likely are multifactorial. Grey seals also exhibit sexual dimorphism with males being up to 1.5 times larger than females, and Moore and Wilson [26] show that for mammal species where males are larger than females, males tend to be more parasitized, possibly due to an increased food intake. It is also notable that parasite prevalence was so high in 2008. This partially can be explained by the sex ratio of animals examined that year, which was highly skewed towards males at 2.3 males per female. In contrast, the mean sex ratio for all other years combined was 1.1 males per female. However, even females had the highest parasitism frequency in 2008 (15.9%) compared with all other years (range of 0–11.3%).

Parasitism also increased significantly with age in the present study (Tables 1 and 2). Absence of young animals likely reflects both dietary habits and our methods to detect parasitic infection. Nursing or newly weaned pups will not be infected until they start consuming fish containing metacercariae. Likewise, our method to detect parasites relies on pathological changes associated with parasitism and length of time to develop these changes (grossly visible cholangiohepatitis, cholangitis and portal lymph node hyperplasia) is unknown. Our detection method will have low sensitivity for acute infections. Still, infection level continued to increase with increasing age and although it began to level off with advanced age, it never declined (Table 1). In many host-parasite systems, parasite infection levels typically reach a peak in a certain age class and then decline, and reasons cited include changes in parasite exposure, increased innate resistance and acquired immunity in the host [27]. Reasons for the absence of this peak in this particular host-parasite system was not elucidated, but the same pattern has been described for infections where parasite-induced mortality is low and acquired protective immunity is partial [28]. Our findings also mirror *P*. *truncatum* infection in otters (*Lutra lutra*) in the United Kingdom where infections accumulate with age [29].

Finally, animals killed in fishery interactions were less likely to be infected than those hunted or found dead or debilitated. Pups were overrepresented in fishery interactions which may play a role in this finding. Additionally, grey seals collected from fishing gear have been shown to have a different diet as compared to seals collected elsewhere in the Baltic Sea [20], so differences in level of parasitism may reflect differences in exposure to fish infected with *P*. *truncatum*. Animals killed in fishery interactions died more often during the autumn, but it is difficult to speculate how this may have impacted the relatively lower proportion of parasitism observed in these seals. Finally, just over 60% of animals that died in fishery interactions came from the Baltic Proper, where salinity is on average higher than in the Gulf of Bothnia. Roach are a freshwater species and salinity levels in the Baltic Proper may limit their distribution. However, in our analyses, sea basin was not found to be significantly associated with parasitism.

Although the majority of hepatobiliary infections were mild to moderate, severe parasitism resulted in liver failure at least in one individual, and probably also in two other seals. To our knowledge, this is the first documentation of severe pathology caused by *P*. *truncatum* infection in grey seals. With the exception of the type specimens reported from the harbour seal (*Phoca vitulina*) [2], the only published reports of *P*. *truncatum* in marine mammals are from Caspian seals (*Pusa caspica*) [7,30,31,32]. Similar pathological changes (chronic cholangitis and cholecystitis with associated hepatic necrosis) and even death have been attributed to this parasite in Caspian seals. Kuiken et al [7] also described pancreatic infection in Caspian seals. Although no involvement of the pancreas or pancreatic ducts was detected in grey seals in this study (n = 1,554), a parasitized grey seal examined in 2015 showed both hepatic and pancreatic infection (Bäcklin, personal observation). We conclude that *P*. *truncatum* only rarely extends into the pancreas of grey seals, but it clearly has the potential to cause significant hepatobiliary damage. As grey seals live in a highly contaminated environment in the Baltic [33], and because the liver is the major detoxification organ, these animals may be particularly vulnerable to impaired hepatic function.

*P*. *truncatum* is emerging in mustelids in the United Kingdom and Ireland and introduction of invasive cyprinid bait fish was hypothesized as a possible source [14]. The life cycle of *P*. *truncatum* in the Baltic ecosystem has not been elucidated, but the roach has been documented as an intermediate host in other areas of northern Europe [3,4,5] and is therefore a primary candidate for an intermediate fish species in the Baltic environment. Roach are now abundant throughout Baltic coastal areas of Sweden north of Skåne and cyprinids as a whole are significantly increasing in the Gulf of Bothnia in the north [34,35]. Diet analysis of grey seals from the Baltic also supports the role of roach or other cyprinids in parasitism. Even though diet analysis using prey hard parts has its limitations and reflects only the most recently ingested prey items, occurrence of cyprinid hard parts in gastrointestinal contents of seals was ten times greater in 2008–2013 compared to 2002–2007, coinciding with the emergence of *P*. *truncatum* in grey seals. Finally, further support for roach as the intermediate host may be inferred from the absence of the parasite in seals collected from the most southern part of Sweden where salinity may limit the distribution of roach and other cyprinids offshore. However, sample size of seals examined from southern Sweden (Skåne and Blekinge counties) was small compared to other areas (Fig 1) and a very limited number of gastrointestinal tracts were available from hunted seals from this area for dietary analysis. Further analyses on seals from southernmost Sweden and targeted investigations to definitively identify the intermediate fish hosts clearly are needed.

Emergence of *P*. *truncatum* may reflect changes in abundance and distribution of the intermediate fish host and changing dietary preferences of seals. Other factors that may influence emergence include conditions favourable for the development and expansion of suitable gastropod intermediate hosts and changes in seal susceptibility. Changing patterns of disease in wildlife often signal changes in the ecosystem, therefore further investigation into reasons for the emergence of *P*. *truncatum* in Baltic grey seals is highly warranted.

In conclusion, *P*. *truncatum* is a generalist that can infect a wide range of mammalian species, where infection may occur through the ingestion of raw or improperly cooked fish containing infective metacercariae. The emergence of this parasite in grey seals signals the relatively common occurrence of *P*. *truncatum* in the Baltic Sea ecosystem. Thus, the risk for and extent of infection of other mammals, including humans, needs to be further evaluated and requires definitive identification of the intermediate fish hosts that harbor the metacercaria.

## Supporting Information

S1 Appendix Grey seal (*Halichoerus grypus*) carcasses and tissues from the Baltic Sea examined by the Swedish Museum of Natural History from 2002–2013. (XLSX)

S2 Appendix Presence of cyprinid hard parts in gastrointestinal tracts of hunted grey seals (*Halichoerus grypus*) from the Baltic Sea examined from 2002–2013. (XLSX)
